# Record of Mortality in the City of Buffalo, for the Month of February, 1854

**Published:** 1854-04

**Authors:** J. M. Newman

**Affiliations:** Health Physician


ART. XII.—*Record of Mortality in the City of Buffalo, for the Month of February*, 1854. By J. M. NEWMAN, Health Physician.

| DISEASES. | No. | Males. | Females. |
|---|---|---|---|
| Albuminuria, | 1 | | 1 |
| Apoplexy, | 3 | 1 | 2 |
| Bronchitis, | 1 | 1 | |
| Bronchitis Typhoid, | 1 | | 1 |
| Child Bed, | 2 | | 2 |
| Cholera Infantum, | 1 | | 1 |
| Consumption, | 18 | 11 | 7 |
| Convulsions, | 16 | 7 | 7 |
| Croup, | 6 | 3 | 3 |
| Cyanosis, | 1 | | |
| Cynanche Tonsillaris Malig., | 1 | | 1 |
| Diarrhœa, | 2 | | 2 |
| Diarrhœa, Chronic, | 1 | 1 | |
| Disease of Kidneys, | 1 | 1 | |
| Dropsy, | 3 | 2 | 1 |
| Dysentery, | 3 | 2 | 1 |
| Fever, | 1 | | 1 |
| " Typhoid, | 8 | 4 | 4 |
| " Typhus, | 1 | 1 | |
| " Puerperal, | 1 | | 1 |
| Hepatitis, | 1 | | 1 |
| Hydrocephalus, | 3 | 1 | 2 |
| Hypertrophy of Heart, | 1 | 1 | |

REGISTER OF MORTALITY—(Continued.)

| DISEASES. | No. | Males. | Females. |
|---|---|---|---|
| Inflammation of Bowels, | 2 | 2 | |
| " " Brain, | 1 | | 1 |
| " " Spinal Marrow, | 1 | 1 | |
| " and ulceration of Mucous Membrane, | 1 | | |
| Injury to Heart, | 1 | 1 | |
| Measles, | 7 | 4 | 3 |
| Meningitis in course of Rheumatism, | 1 | | 1 |
| Old Age, | 7 | 5 | 2 |
| Pneumonia, | 5 | 2 | 3 |
| " Typhoid, | 2 | 1 | 1 |
| Paralysis, | 1 | | 1 |
| Pleurisy, | 1 | | 1 |
| Purpura Hæmorrhagica, | 1 | | 1 |
| Scalded, | 1 | | 1 |
| Scarlatina, | 10 | 3 | 6 |
| Small-pox, | 2 | 1 | 1 |
| Still-born, | 4 | | |
| Suffocation from enlarged Cervical Glands, | 1 | 1 | |
| Teething, | 1 | | 1 |
| Unknown, | 13 | 6 | 5 |
| | 140 | | |

| | |
|---|---|
| Males, | 64 |
| Females, | 66 |
| Sex not given, | 10 |
| Total, | 140 |

AGES.

| | | | |
|---|---|---|---|
| Still-born, | 4 | Between 3 years and 6 years, | 15 |
| 1 day, | 2 | " 6 " " 12 " | 4 |
| Between 1 day and 15 days, | 12 | " 12 " " 20 " | 7 |
| " 15 days and 30 days, | 2 | " 20 " " 30 " | 18 |
| " 1 month and 3 months, | 2 | " 30 " " 40 " | 9 |
| " 3 months and 6 months, | 7 | " 40 " " 50 " | 8 |
| " 6 " " 9 " | 9 | " 50 " " 60 " | 7 |
| " 9 " " 12 " | 7 | " 60 " " 70 " | 5 |
| " 1 year and 3 years, | 17 | Ages not given, | 5 |
| Total, | | | 140 |

NATIVITY.

| | | | |
|---|---|---|---|
| American, | 51 | French, | 1 |
| English, | 1 | Swiss, | 2 |
| Scotch, | 2 | Pole, | 1 |
| Irish, | 10 | Colored, | 2 |
| German, | 39 | Country not given, | 30 |
| Canadian, | 1 | | |
| Total, | | | 140 |

From the above tables it will be observed that many of the certificates are still defective in not giving the sex and nation of the deceased.

During the month two sextons were detected, prosecuted and fined, for making interments without "permits."

